# Circadian disturbances, anxiety and motor disturbances differentiate delirium superimposed on dementia from dementia-only

**DOI:** 10.3389/fpsyt.2024.1407213

**Published:** 2024-08-22

**Authors:** Thiemo Schnorr, Tim Fleiner, Rieke Trumpf, Christian Prüter-Schwarte, Janina Fanselow, Wiebren Zijlstra, Peter Haussermann

**Affiliations:** ^1^ Department of Geriatric Psychiatry and Psychotherapy, LVR-Hospital Cologne, Cologne, Germany; ^2^ Institute of Movement and Sport Gerontology, German Sport University Cologne, Cologne, Germany; ^3^ Institute for Geriatric Research, Ulm University Medical Centre, Ulm, Germany; ^4^ Department of Forensic Psychiatry and Psychotherapy, LVR-Hospital Cologne, Cologne, Germany

**Keywords:** delirium superimposed on dementia, motor subtypes, acute hospital, neuropsychiatric symptoms, sleep disturbances

## Abstract

**Background:**

To ensure adequate treatment, individuals with delirium superimposed on dementia (DSD) need to be differentiated reliably from those with dementia only (DO). Therefore, we aimed to examine the clinical indicators of DSD by assessing motor subtypes, cognitive performance and neuropsychiatric symptoms in DSD and DO patients.

**Methods:**

Cross-sectional design with the Delirium-Motor-Subtyping Scale (DMSS), Mini-Mental-State-Examination (MMSE), Clock-Drawing-Test (CDT), DemTect, and Neuropsychiatric Inventory assessed after admission to an acute hospital.

**Results:**

94 patients were included, 43 with DSD (78 ± 7 years, MMSE = 11 ± 9) and 51 with DO (79 ± 7 years, MMSE = 9 ± 8). DMSS “no subtype” was more common in the DO group (26% vs. 10%, *p* = .04). The DSD group showed lower CDT scores (*DSD: M =* 4 ± 3 vs. DO: M = 6 ± 1; *p* < .001) and higher anxiety (DSD: *MED* = 3 ± 8 vs. DO: *MED* = 3 ± 4; *p* = .01) and sleep/night-time behavior disturbances (DSD: *MED* = 0 ± 6 vs. DO: *MED* = 0 ± 0; *p* = .02).

**Conclusions:**

Sleep/night-time behavior disturbances appear to be a clinical indicator of DSD. Motor subtypes can identify cases at increased risk of developing delirium or unrecognized delirium.

**Clinical trial registration:**

https://drks.de/search/de/trial/DRKS00025439, **identifier DRKS00025439**.

## 1 Introduction

Delirium is one of the most common neuropsychiatric disorders in hospitalized older adults and is characterized by acute disturbances of attention, awareness, and cognition (1). Delirium in the presence of pre-existing dementia is termed delirium superimposed on dementia (DSD) and has been reported in over 50% of hospitalized older adults, with increasing incidence rates as dementia severity progresses (2, 3). DSD is associated with increased costs to healthcare systems, higher rehabilitation needs, and caregiver burden (4), as well as in-hospital mortality rates of 25% to 33% (5, 6) and increased post-discharge mortality (7, 8).

As the treatment for and outcome of DSD differs from that of dementia only (DO) (9, 10), early and appropriate recognition of DSD is crucial to enable prompt and effective neuroleptic treatment, which may prevent poorer outcomes in patients with DSD (11). Indeed, research has shown that delirium remains unrecognized in up to 72% of hospitalized older adults (12). Additionally, several studies have revealed a high occurrence of disagreement between the subjective assessment of delirium by clinicians, intensivists, and nurses in acute care settings and assessments conducted by research teams using validated and reliable delirium assessment tools, such as the Confusion Assessment Method (CAM) (13). While the specificity of delirium detection by medical staff has been reported to be 100% in several studies, misdiagnosis rates are also consistently high, ranging from 65% to 72% (14, 15). Notably, misdiagnosis is especially common in settings without structured delirium assessment instruments, and the most significant risk factors for the under-recognition of delirium are hypoactive delirium, age of 80 years and older, vision impairment, and comorbid dementia (16).

The overlap of cognitive, behavioral and psychological symptoms observed in both dementia and delirium complicates the proper recognition and identification of DSD (17). For this reason, established diagnostic tools such as the International Statistical Classification of Diseases and Related Health Problems, 10^th^ Revision (ICD-10) (18) and the Diagnostic and Statistical Manual of Mental Disorders, Fifth Edition (DSM-5) (19) do not provide clinicians with sufficient specific criteria to ensure accurate recognition and diagnosis of DSD (20). In addition to the missing specificity in these guidelines, there is a high heterogeneity in both delirium assessment methods and delirium symptom termination, further complicating delirium diagnosis (21). However, there is a need to identify indicators that may help to adequately identify delirium and differentiate hospitalized patients with DSD from those with DO, particularly in the acute hospital setting. In addition to supporting early and appropriate delirium management, clinical practice would benefit from increased knowledge of the phenotypes, underlying mechanisms, and individual symptoms of DSD vs. DO, as well as improvements in individualized treatment and under-recognition of delirium.

Several studies have suggested that differences in cognitive domains (i.e., attention, awareness, and arousal) (22, 23) and neuropsychiatric symptoms (NPS; i.e., sleep-wake disturbances, affective lability, and motor activity changes) (24–26) may be possible indicators of DSD. However, these findings should be considered cautiously, as outcomes in terms of cognitive domains and NPS in patients with DSD and DO vary widely between settings and samples. Indeed, dementia alone affects both domains prior to the possible onset of delirium, highlighting the challenge of differentiating DSD from DO in acute hospital settings, especially when considering individuals with advanced dementia (21).

In addition to the above indicators, another promising approach to identifying DSD is the assessment of psychomotor disturbances (27). Disturbed motor behavior is characteristic of patients who have developed delirium and can be categorized into four motor subtypes: hypoactive (lethargy/inactivity), hyperactive (agitation), mixed, and no subtype (28). In particular, the hypoactive subtype is associated with a significantly higher rate of mortality as compared to the hyperactive and mixed subtypes (29, 30). From a clinical and prognostic perspective, this higher mortality observed for the hypoactive subtype highlights the risks of unrecognized delirium. Moreover, motor fluctuations may act as an indicator of delirium development. Therefore, the assessment of motor subtypes provides an opportunity not only to ease the identification of delirium but also to detect patients at increased risk of developing delirium (31, 32).

Overall, there is a need for more reliable detection of delirium and for indicators that differentiate between DSD and DO patients, particularly in vulnerable samples, such as in acute hospital settings. Due to the lack of knowledge on this topic and inconsistencies in results, the aims of this study are as follows: (a) to assess motor subtypes, cognitive performance, and neuropsychiatric symptoms in patients with DSD and DO, and (b) to investigate group differences in these characteristics.

## 2 Methods

### 2.1 Study design and sample

A cross-sectional, monocentric study was conducted at the Department of Geriatric Psychiatry of the LVR Hospital Cologne, Cologne, Germany, from June 1, 2021, to June 30, 2022. The department includes secure and open wards and provides acute care for geriatric patients with psychiatric disorders who are experiencing an acute threat to their health, i.e. are severely ill in terms of psychiatric or other medical condition. Patients admitted to an acute hospital for geriatric psychiatric treatment are often at acute risk of harming themselves or others. Referrals to the LVR Hospital can be made by general practitioners, hospitals, the police or on a voluntary basis, with or without pre-existing clinical diagnosis of dementia or delirium. Upon admission, trained physicians and psychologists evaluate each patient and confirm existing diagnoses or make new diagnoses according to ICD-10 criteria and the Confusion Assessment Method (CAM) Diagnostic Algorithm for delirium. The CAM includes four features found to have the greatest ability to distinguish delirium from other types of cognitive impairment: (1) Acute onset or fluctuating course; (2) Inattention; (3) Disorganized thinking; (4) Altered level of consciousness. Delirium was diagnosed when features 1 and 2 and either 3 or 4 were present. Patients are discharged from the LVR Hospital once their psychiatric and geriatric medical conditions have stabilized (e.g., remission of delirium). All patients with a diagnosis of dementia or DSD were screened for eligibility for this study. Written informed consent was obtained from all the included patients or their legal guardians. Cases were included according to the following criteria: (a) age 60 years or older, (b) diagnosis of dementia or DSD according to the ICD-10 criteria as assessed by the CAM, and (c) written consent of the legal guardian. The exclusion criteria were as follows: (a) no legal guardian, (b) immobile/bedridden, and (c) no consent to participate.

### 2.2 Outcome measures

Outcome measures were: (a) motor subtypes, (b) neuropsychiatric symptoms, and (c) cognitive performance. The respective assessments were administered at a single time point within the first 24 to 48 hours after admission. While the research team assessed motor subtypes and neuropsychiatric symptoms independently, cognitive performance data were extracted from the standardized comprehensive geriatric assessment. This assessment battery is administered within the first 24 hours of each admission to the LVR Hospital Cologne by trained medical and psychological staff and assesses several domains of clinical relevance, such as cognitive and physical performance, activities of daily living and risk of falls. The comprehensive geriatric assessment is partially repeated at discharge, but for this cross-sectional study only the data from the first measurement time were analyzed. In addition, demographic and medical data were extracted from the hospital information system. This information included the sex, age, body mass index (BMI), ICD-10 diagnosis, and physical and cognitive performance assessment.

#### 2.2.1 Motor subtypes

Motor subtypes were identified using the Delirium Motor Subtyping Scale (DMSS) (33). The DMSS is a proxy assessment of motor disturbance that classifies subjects into four motor subtypes: (1) hyperactive, (2) hypoactive, (3) mixed, and (4) no subtype. The scale consists of 11 items (four for hyperactive and seven for hypoactive) and is administered to relatives, caregivers, or clinicians who are in close contact with the individual. If two or more items from the hyperactive or hypoactive subtypes are present, a certain motor subtypes can be identified. If subjects have one or no hypomotoric or hypermotoric features, they are classified as (4) no subtype.

#### 2.2.2 Cognitive performance

The cognitive assessments used in this study included the Mini-Mental-State Examination (MMSE) (34), the Clock Drawing Test (CDT) (35), and the DemTect (36).

Firstly, the MMSE is a validated 30-item questionnaire that measures cognitive function and takes a relatively short time to complete (5–10 minutes). The MMSE assesses the severity of deficits in several cognitive functions, including orientation, registration, attention, recall, language, and the ability to follow simple commands. Correct answers or correctly performed tasks are worth 1 point, resulting in a total score between 0 and 30. Defined cut-off values are used to indicate the severity of cognitive impairment measured by the MMSE, with ≥24 points indicating normal cognition, 19–23 points indicating mild cognitive impairment, 10–18 points indicating moderate cognitive impairment and ≤9 points indicating severe cognitive impairment.

The CDT is a quick and easy-to-administer instrument used to assess cognitive functioning in terms of verbal comprehension, perceptual and executive functions, motor coordination, concentration, spatial knowledge, visuospatial ability, and verbal and semantic memory. Scores on the CDT are independent of education, ethnic group, socio-economic status, and language effects and are reported to be sensitive to changes in cognitive function, with good predictive validity and inter-rater reliability (37). For this assessment, the subjects receive a pencil and a sheet of paper with a circle on it. The circle is presented as an “incomplete clock”, and the subject is asked to complete the clock to show a time of 11:20 (standardized in this study). According to Shulman et al., the results of the CDT can be scored on a 6-point scale, with 1 point representing the best score (1 = perfect clock; 2 = minor visuospatial errors; 3 = correct clock, wrong time; 4 = moderate deficits in visuospatial orientation; 5 = severe deficits in visuospatial orientation; 6 = no clock drawn/drawing does not resemble a clock) (38).

The DemTect is a short (8–10 minutes) and easy-to-administer test for the assessment of cognitive impairment. Specifically, the DemTect comprises five tasks, including remembering a word list, a number transcoding task, a semantic verbal fluency task, reversed digit span, and delayed recall of the word list. The scores from the different tasks are converted into a total score between 0 and 18 points. Based on the work of Kalbe et al. (36), the cut-off scores for subjects over the age of 60 were defined as follows: 13–18 points = age-appropriate cognitive performance; 9–12 points = mild cognitive impairment; ≤8 points = dementia should be suspected.

#### 2.2.3 Neuropsychiatric symptoms

In this study, neuropsychiatric symptoms and related caregiver burden were assessed using the Neuropsychiatric Inventory Questionnaire (NPI) (39). The NPI assesses the development of neuropsychiatric symptoms over a defined period of time (maximum of 1 month). The NPI covers 12 dimensions (delusions, hallucinations, agitation/aggression, depression/dysphoria, anxiety, elation/euphoria, apathy/Indifference, disinhibition, irritability/lability, aberrant motor activity, sleep/night-time behavioral issues and eating abnormalities) and is administered to the subject’s caregiver. In this study, the respondents were asked about whether symptoms from of each sub-dimension had occurred since admission and. If the answer was “yes”, the frequency (1 = rarely, 2 = sometimes, 3 = often, 4 = very often), severity (1 = mild, 2= moderate, 3 = severe), and burden (0 = not at all, 1 = fractional, 2 = slight, 3 = moderate, 4 = severe, 5 = extreme) of the symptom was then assessed. For this scale, the score for each domain is calculated by multiplying the frequency by the severity of each symptom. The sum of the 12 symptom scores represents the total symptom expression score, while the sum of the burden of each domain gives the total caregiver burden score. The DMSS and NPI assessments were conducted within 48 hours of admission at a single time point by TS (*clinical researcher*) and JF (*medical doctor-in-training)*, who were trained by a senior clinician.

### 2.3 Statistical analysis

Data were analyzed using the IBM Statistical Package for the Social Sciences (SPSS) 29 for Windows (IBM Corporation, Route, Somers, NY, USA). Descriptive analysis of the demographic and clinical characteristics of the participants was performed using frequencies and proportions, means and standard deviations, and medians and interquartile ranges. The Shapiro-Wilk test was used to test for normal distribution. Group comparisons were performed using the chi-squared test or Fisher’s exact test for categorical data, the t-test for continuous and normally distributed data, and the Mann-Whitney U test for non-parametric or non-normally distributed data. All statistical tests were two-tailed and p-values <0.05 were considered to represent statistical significance.

## 3 Results

**Figure 1:**
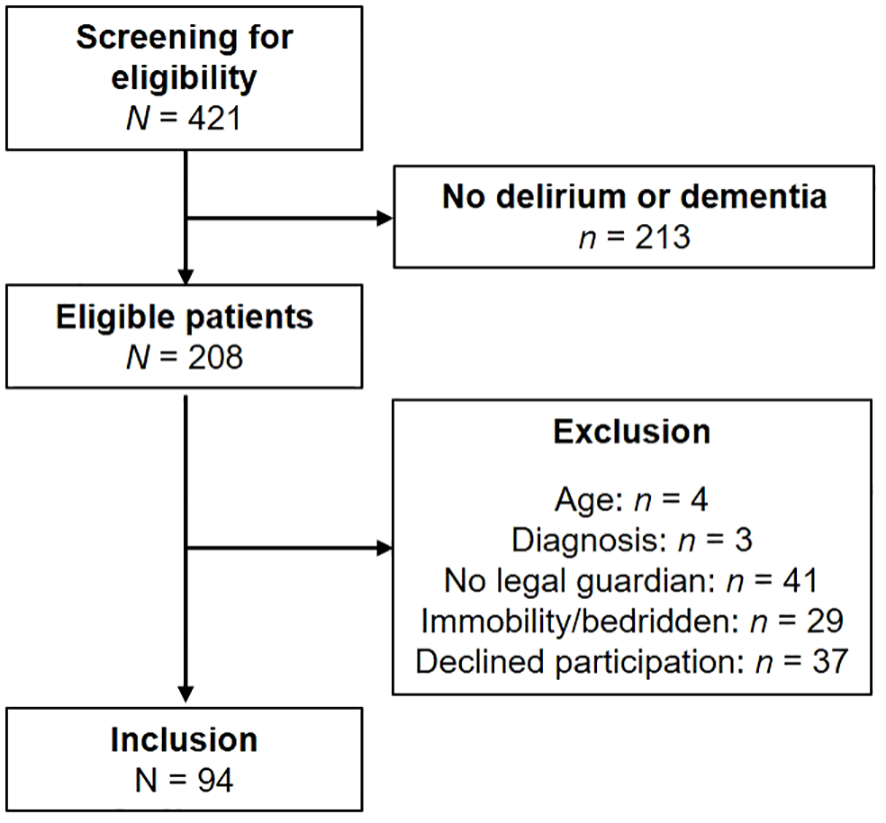
Study flowchart.

From June 1, 2021 to June 30, 2022, *N* = 421 patients were admitted to the included wards of the geriatric psychiatry department of the LVR-Hospital Cologne, of whom *N* = 208 were diagnosed with DSD or dementia. After screening for eligibility, *N* = 94 patients were included in the data analysis (**Figure 1**). The mean age of the total sample was 79 years (*SD* = 7), with 43 patients with DSD (mean age: 78 years; *SD* = 7) and 51 patients with dementia (mean age: 79 years; *SD* = 7). The DSD group comprised 32 (74%) patients with mixed type dementia, 8 (19%) patients with Alzheimer’s dementia, and 3 (7%) patients with vascular dementia. In the DO group, mixed type dementia was present in 33 (65%) patients, Alzheimer’s dementia in 14 (27%) patients, and vascular dementia in 4 (8%) patients. Analyses of the demographic data from the standardized comprehensive geriatric assessment showed no differences between the patients with DSD and DO except for in terms of their CDT total scores (*M* = 4, *SD* = 3 vs. *M* = 6, SD = 1; *p* < .001; **Table 1**). Due to affective and behavioral disturbances, 18 patients were unable to complete the cognitive assessment, including 12 (28%) patients from the DSD group and 6 (12%) from the DO group (*p* = .04).

**Table 1:** Clinical characteristics of the total sample and according to delirium and dementia diagnoses.

	Total sample (*N* = 94, 55% female)				DSD (*n* = 43; 58% female)			DO (*n* = 51; 53% female)			Mean Diff. (t-test/M-W)		
	*N (%)^a^*	*M (SD)^b^*	*Min*	*Max*	*M (SD)*	*Min*	*Max*	*M (SD)*	*Min*	*Max*	*t/Z^d^*	*df*	*p^e^*
Age [years]		78 (7)	62	98	78 (7)	64	98	79 (7)	62	94	0.72	92	.47
BMI [kg/m²]		24 (4)	15	36	24 (3)	16	36	23 (4)	15	34	0.49	88	.62
TUG [sec]	39 (41)	15 (8)*^c^*	9	37	18 (8)	8	37	17 (6)	10	31	-0.36*^f^*	-	.72
SPPB^g^	84 (89)	5 (9)	0	12	4 (9)	0	12	6 (8)	0	12	-0.87	82	.39
MMSE^h^	76 (81)	10 (8)*^c^*	0	24	11 (9)	0	24	9 (8)	0	23	-1.31*^f^*	-	.19
CDT^i^	76 (81)	6 (2)*^c^*	1	6	5 (4)	1	6	6 (1)	2	6	-3.53*^f^*	-	**<.001**
DemTect^j^	76 (81)	4 (4)*^c^*	0	15	2 (10)	0	15	2 (7)	0	13	-1.61*^f^*	-	.09
NPI^k^		25 (16)	0	68	28 (17)	0	68	22 (14)	0	65	1.67	92	.10
NPI burden^l^		9 (6)	0	27	10 (6)	0	27	8 (6)	0	27	1.90	89	.06
DMSS subtype		*N (%)*			*N (%)*			*N (%)*			*χ²*	*p*	
Hyperactive		38 (40)			21 (46)			17 (32)			*2.328*	.13	
Hypoactive		26 (28)			12 (28)			14 (28)			*0.002*	.96	
Mixed		13 (13)			6 (14)			7 (14)			*0.001*	.96	
No subtype		17 (19)			4 (10)			13 (26)			*4.127*	**.04**	

^a^ assuming complete sample size unless otherwise indicated. ^b^ patient characteristics are presented as the mean (M) and standard deviation (SD) unless stated otherwise. ^c^ Median (MED) and interquartile range (IQR) for not normally distributed data. ^d^ t-test used for normally distributed data, non-parametric test (Mann-Whitney U-test) used for not normally distributed data. ^e^ Significant results highlighted in bold.

^f^ data not normally distributed. ^g^ range: 0–12 points (0 meaning severe deficits in mobility). ^h^ range: 0–30 points (0 meaning severe symptoms). ^i^ range: 1–6 points (0 meaning severe deficits). ^j^ range: 0–15 points (0 meaning severe deficits). ^k^ NPI total score, range 0–144 points (0 meaning absence of symptoms). ^l^ range 0–48 points (0 meaning no caregiver burden). DSD, delirium superimposed on dementia; DO, dementia only; BMI, body mass index; TUG, Timed “Up & Go” Test; SPPB: Short Physical Performance Battery; MMSE, Mini Mental State Examination; CDT, Clock Drawing Test; NPI, Neuropsychiatric Inventory; DMSS, Delirium Motor Subtyping Scale; M-W, Mann-Whitney U-test. “-”, degrees of freedom not applicable in Mann-Whitney U-test.

**Figure 2:**
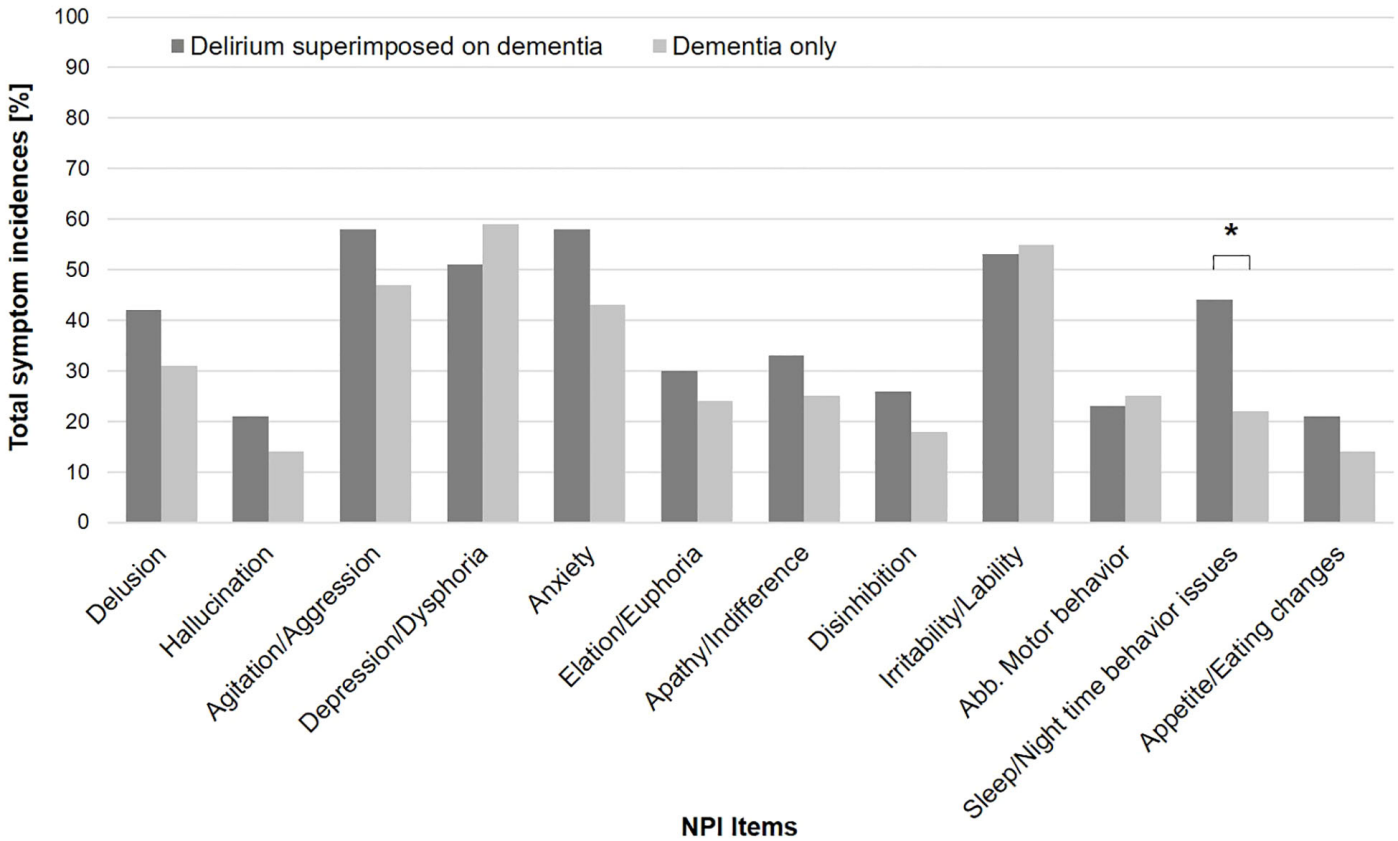
Total NPI incidence in DSD and DO group. *Delirium superimposed on dementia > dementia only at p <. 05.

The distribution of DMSS motor subtypes is presented in **Table 1**. In the total sample, as well as in the DSD and DO subgroups, the hyperactive subtype was the most common, followed by the hypoactive subtype. A chi-squared analysis of group distribution showed a significant difference in the distribution of motor subtypes within the patients with DSD and DO (*p* = .04), as there were significantly more patients with no subtype in the DO group compared to the DSD group (26% vs. 10%; *p* = .04). **Figure 2** shows the total symptom incidence for the 12 NPI items in the DSD and DO groups, with a significantly higher incidence of sleep/night-time behavior issues in the DSD group compared to the DO group (44% vs. 22%; *p* = .02).

For the NPI items anxiety (DSD: *MED* = 3, *IQR* = 8 vs. DO: *MED* = 3, *IQR* = 4; *p* = .01) and sleep/night-time behavior issues (DSD: *MED* = 0, *IQR* = 6 vs. DO: *MED* = 0, *IQR* = 0; *p* = .02), the DSD group showed higher symptom severity scores (**Table 2**). Regarding the NPI burden, the DSD group also showed higher scores for the items anxiety (DSD: *MED* = 1, *IQR* = 2 vs. DO: *MED* = 0, *IQR* = 1; *p* = .03) and sleep/night-time behavior issues (DSD: *MED* = 0, *IQR* = 3 vs. DO: *MED* = 0, *IQR* = 0; *p* = .02).

**Table 2:** Neuropsychiatric symptoms measured by the NPI for both groups.

NPI total score	DSD (n = 43)			DO (n = 51)			Mean Diff.	
	*MED* (*IQR)*	*MIN*	*MAX*	*MED* (*IQR)*	*MIN*	*MAX*	*Z* ^a^	*p* ^b^
Delusion	0 (6)	0	12	0 (4)	0	12	-1.02	.31
Hallucination	0 (0)	0	12	0 (0)	0	12	-0.96	.34
Agitation/Aggression	2 (6)	0	12	0 (6)	0	12	-0.90	.37
Depression/Dysphoria	2 (6)	0	12	3 (6)	0	12	-0.62	.53
Anxiety	3 (8)^c^	0	12	3 (4)	0	12	-1.71	**.01**
Elation/Euphoria	0 (2)	0	8	0 (0)	0	8	-0.36	.72
Apathy/Indifference	0 (4)	0	8	0 (2)	0	12	-0.80	.42
Disinhibition	0 (1)	0	12	0 (0)	0	6	-1.04	.30
Irritability/Lability	1 (8)	0	12	1 (8)	0	12	-0.42	.67
Abb. Motor behavior	0 (0)	0	12	0 (1)	0	12	-0.12	.90
Sleep/Night-time behavior issues	0 (6)^c^	0	12	0 (0)	0	12	-2.40	**.02**
Eating abnormalities	0 (0)	0	9	0 (0)	0	12	-0.99	.32
NPI Burden								
Delusion	0 (2)	0	4	0 (2)	0	5	-0.84	.40
Hallucination	0 (0)	0	3	0 (0)	0	4	-0.97	.33
Agitation/Aggression	2 (3)	0	5	0 (3)	0	5	-0.73	.46
Depression/Dysphoria	0 (2)	0	3	1 (2)	0	3	-0.85	.40
Anxiety	1 (2)^c^	0	4	0 (1)	0	3	-2.21	**.03**
Elation/Euphoria	0 (0)	0	2	0 (0)	0	3	-0.08	.93
Apathy/Indifference	0 (1)	0	2	0 (0)	0	3	-0.82	.41
Disinhibition	0 (1)	0	5	0 (0)	0	5	-0.86	.39
Irritability/Lability	2 (3)	0	4	2 (3)	0	4	-0.71	.94
Abb. Motor behavior	0 (0)	0	3	0 (1)	0	5	-0.04	.97
Sleep/Night-time behavior issues	0 (3)^c^	0	5	0 (0)	0	4	-2.55	**.01**
Eating abnormalities	0 (0)	0	3	0 (0)	0	2	-1.01	.31

^a^ Mann-Whitney U-test due to not normally distributed data. ^b^ Significant results highlighted in bold. ^c^ DSD > DO at p <.05.

DSD, Delirium superimposed on dementia; DO, dementia only; MED, median; IQR, Interquartile range; MIN, Minimum; MAX, Maximum; NPI, Neuropsychiatric Inventory.

## 4 Discussion

The aims of this study were as follows: (a) to assess motor subtypes, cognitive performance, and neuropsychiatric symptoms in patients with DSD and DO, and (b) to investigate group differences in these characteristics. The main results of this analysis showed that, compared to the DSD group, the DO group had a higher proportion of patients classified as “no subtype” on the DMSS. In addition, patients with DSD showed significantly lower scores on the CDT, greater severity of anxiety and sleep/night-time behavior issues on the NPI, and greater caregiver burden on both measures.

Motor fluctuations are considered to be a very specific finding in the spectrum of delirium states, thus facilitating the differentiation between DSD and DO patients (40). However, to date, this is the first study to assess DMSS motor subtypes in patients with DSD compared with patients with DO in an acute hospital setting. Previous studies investigating motor fluctuations and disturbances in delirium and dementia have found more severe disturbances in DSD compared to DO (41). Moreover, the “no subtype” classification on the DMSS has been observed to be more common in samples of patients without delirium but with related neuropsychiatric presentations compared to DSD and delirium only patients (42). Notably, the results of our study support these findings. However, the distribution of the remaining motor subtypes (hyperactive, hypoactive, and mixed subtypes) in this sample showed no differences between the two groups. Therefore, aberrant motor behavior, either hypo- or hyperactive, may indicate delirium, but the assessment of motor behavior by the DMSS lacks specificity for distinguishing delirium from dementia. Nevertheless, it seems relevant to raise awareness and train clinical staff or caregivers to consider hypo- and hyperactive motor behavior as potential risk factors for the development of delirium and thus initiate early delirium assessment and support adequate delirium management.

However, the high proportion of patients with hyperactive and hypoactive subtypes in the DO group in our study (N = 61, 60%) is of high clinical relevance. Given that motor fluctuations are associated with delirium and that clinicians are at high risk of missing delirium cases (43, 44), especially when dementia is comorbid (45) and a hyperactive subtype is present (46), we may have misdiagnosed cases of DSD in our DO group, thus biasing our results. However, as patients are screened for delirium on admission to this setting by trained psychiatrists using the CAM, the risk of missed delirium diagnoses in this study should be lower than in settings that are not specialized in neuropsychiatric disorders. Nevertheless, based on the results of previous studies, the presence of aberrant motor behavior in the DO group may indicate an increased risk of developing delirium in at least 60% of the patients with DO, thus emphasizing the importance of continuous screening for delirium during hospitalization.

Aside from motor fluctuations, previous investigations into the assessment of cognitive performance and neuropsychiatric profiles in patients with DSD compared with DO have shown inconsistent results due to variations in delirium expression, dementia severity, and comorbidities in different samples and settings. Moreover, to date, the existing studies have not been able to elucidate whether cognitive performance on assessments such as the MMSE, CDT, and DemTect is reflective of the severity of cognitive impairment alone or could be related to the manifestation of delirium symptoms (47).

This study revealed significant differences in CDT scores between the DSD and DO groups (with lower CDT scores in patients with DSD compared to DO), but there were no differences in MMSE and DemTect scores. However, recent research has shown that the CDT lacks specificity and sensitivity for detecting delirium in comorbid dementia and that cognitive tests covering multiple cognitive domains, such as the MMSE, may be more appropriate (48). Therefore, our study confirms that cognitive performance assessments in DSD show inconsistent results and may not be useful for detecting delirium in samples and settings where the severity of dementia is high.

As noted above, the results of studies investigating the neuropsychiatric symptoms observed in patients with DSD and DO vary widely. Notably though, in line with the results of the present study, previous studies are consistent in showing that sleep/night-time behavior disturbances are more common in patients with DSD compared with DO (24, 25, 49, 50). However, the higher level of anxiety in patients with DSD found in this study is not consistent with previous findings comparing DSD and DO, but confirms studies that have examined patients with delirium alone (51, 52). This underlines the heterogeneity in terms of study setting and sample characteristics in DSD and DO research, but also emphasizes the individuality of delirium. In addition, other neuropsychiatric characteristics, such as motor agitation and affective lability (24, 49) or aggression (50), have been found to be higher in patients with DSD compared with DO, but these characteristics did not differ between the two groups in this study. The incidence of multiple neuropsychiatric symptoms in this sample may be explained by the high severity of dementia independently of delirium and must be considered a risk factor for delirium in individuals with DO (26, 50).

### 4.1 Limitations

The interpretation of the results should consider the following limitations. In particular, the DMSS has not been validated for use in an acute hospital sample with patients with DO. In this study, the DMSS demonstrated its applicability in a non-delirious sample by detecting motor fluctuations and, thus, putative cases of unrecognized delirium or cases at increased risk of developing delirium. These findings need to be verified by future research and validated using objective measurements of motor behavior. Furthermore, motor subtypes should be continuously assessed during hospitalization, as patients with delirium may fluctuate between different motor subtypes (53). Another limitation of this study is that 28% of the patients with DSD did not complete the cognitive assessment, and this number was significantly different from that in the DO group (12%). These patients did not complete the assessment due to relevant neuropsychiatric symptoms (i.e., acute affective or cognitive disturbances). Therefore, and due to the small size of the emerging subgroups, interactions between outcome parameters and underlying dementia or dementia severity were not analyzed, but should be adressed in future research. Finally, the high number of patients who either declined to participate (n = 37) or did not have a legal guardian (n = 41) may have biased the overall characteristics of this sample. However, this could not be definitively confirmed because data from nonconsenting patients were not evaluated or analyzed in accordance with ethical principles.

### 4.2 Conclusion

The present study confirms the findings of the current literature by identifying clinical indicators that may help to better differentiate DSD from DO. Specifically, sleep/night-time behavior disturbances were found to be a neuropsychiatric indicator for delirium in dementia, consistent with the findings of previous studies, and anxiety was also found to indicate DSD. In samples with high dementia severity, such as an acute hospital setting, the DMSS can detect motor fluctuations and may therefore be helpful in identifying individuals with dementia at increased risk of developing delirium or even cases of unrecognized delirium.

However, it still remains difficult to distinguish patients with DSD from those with DO. This difficulty is most likely due to the wide variation in delirium, the expression of delirium symptoms, and the severity of dementia in different samples and settings. Therefore, this study emphasizes the need for continuous and structured assessments of delirium and suggests that the assessment of motor behavior should be included in patient screening on admission and during hospitalization. To support the assessment of motor fluctuations, the use of wearable devices such as motion sensors or accelerometers should be considered, as they can provide objective feedback on motor behavior and have been shown to be feasible in samples of patients with delirium (54, 55). Based on patients’ motor activity patterns, intervention strategies can then be initiated to address detrimental motor behavior (e.g., prolonged periods of physical inactivity or nocturnal restlessness), support delirium management, or reduce the risk of developing delirium.

There is a need to raise awareness of poorer cognitive, neuropsychiatric, and motor outcomes as risk factors for delirium in dementia and to train medical staff to recognize these symptoms in sequence. The early and reliable detection of delirium allows for prompt and effective neuroleptic treatment and may therefore prevent poorer outcomes in patients with DSD compared to DO.

## Data Availability

The raw data supporting the conclusions of this article will be made available by the authors, without undue reservation.
